# Electrochemistry of Flavonoids: A Comprehensive Review

**DOI:** 10.3390/ijms242115667

**Published:** 2023-10-27

**Authors:** Ana-Maria Chiorcea-Paquim

**Affiliations:** 1 Instituto Pedro Nunes (IPN), 3030-199 Coimbra, Portugal; anachior@ipn.pt; 2 University of Coimbra, Centre for Mechanical Engineering, Materials and Processes (CEMMPRE), Advanced Production and Intelligent Systems (ARISE), Department of Chemistry, 3004-535 Coimbra, Portugal

**Keywords:** flavonoids, polyphenols, phenolic compounds, electrochemistry, electrochemical sensing, antioxidant

## Abstract

Flavonoids represent a large group of aromatic amino acids that are extensively disseminated in plants. More than six thousand different flavonoids have been isolated and identified. They are important components of the human diet, presenting a broad spectrum of health benefits, including antibacterial, antiviral, antimicrobial, antineoplastic, anti-mutagenic, anti-inflammatory, anti-allergic, immunomodulatory, vasodilatory and cardioprotective properties. They are now considered indispensable compounds in the healthcare, food, pharmaceutical, cosmetic and biotechnology industries. All flavonoids are electroactive, and a relationship between their electron-transfer properties and radical-scavenging activity has been highlighted. This review seeks to provide a comprehensive overview concerning the electron-transfer reactions in flavonoids, from the point of view of their in-vitro antioxidant mode of action. Flavonoid redox behavior is related to the oxidation of the phenolic hydroxy groups present in their structures. The fundamental principles concerning the redox behavior of flavonoids will be described, and the phenol moiety oxidation pathways and the effect of substituents and experimental conditions on flavonoid electrochemical behavior will be discussed. The final sections will focus on the electroanalysis of flavonoids in natural products and their identification in highly complex matrixes, such as fruits, vegetables, beverages, food supplements, pharmaceutical compounds and human body fluids, relevant for food quality control, nutrition, and healthcare research.

## 1. Introduction

Flavonoids are naturally occurring bioactive compounds in plants that act at the physiological level in coloring, aroma, flavor, maturation, and protection against infections and predators. Flavonoids play a vital role in the food, medical, pharmaceutical, cosmetic and biotechnology fields, due to their numerous pharmacotherapeutical properties, such as antibacterial, antiviral, antineoplastic, anti-inflammatory, antiallergic, vasodilatory and cardioprotective actions, among others [1,2]. It has been demonstrated that flavonoids present protective effects against oral, gastro-intestinal, colorectal, liver, reproductive, breast, and lung carcinogenesis [3], and their biomolecular mechanisms may include antioxidant effects produced by the inactivation of reactive oxygen species (ROS) and reactive nitrogen species (RNS), the binding of electrophiles, the induction of protective enzymes, the inhibition of lipid peroxidation, increasing the apoptosis rate, the inhibition of cellular proliferation, angiogenesis inhibition, H-donation, and the inhibition of DNA oxidation.

**Figure 1:**
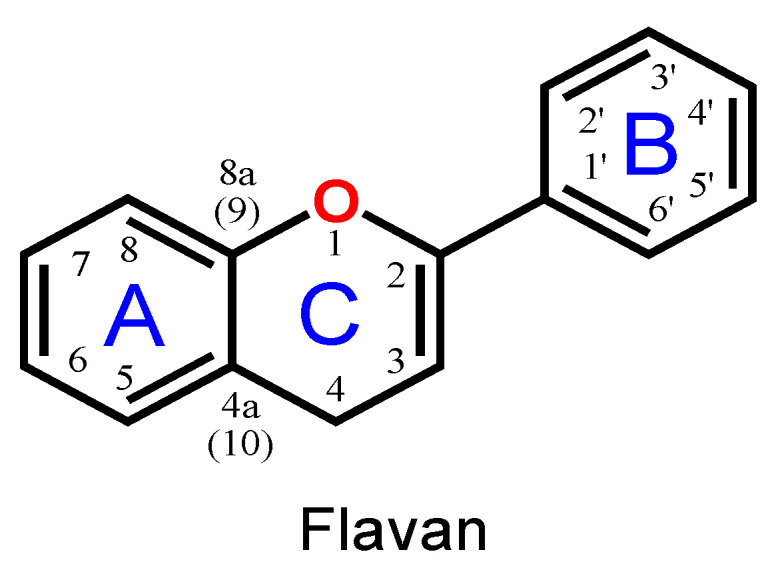
Flavan chemical structure, the central unit of flavonoids, with the A, B and C aromatic rings highlighted.

Flavonoids are phenolic compounds belonging to the class of plant secondary metabolites. Their chemical structure is based on the flavan structure that contains two aromatic rings A and B, linked together by an oxygenated heterocycle C ring, also presenting different hydroxy (–OH), methoxy and glucosides substituents (Figure 1).

**Figure 2:**
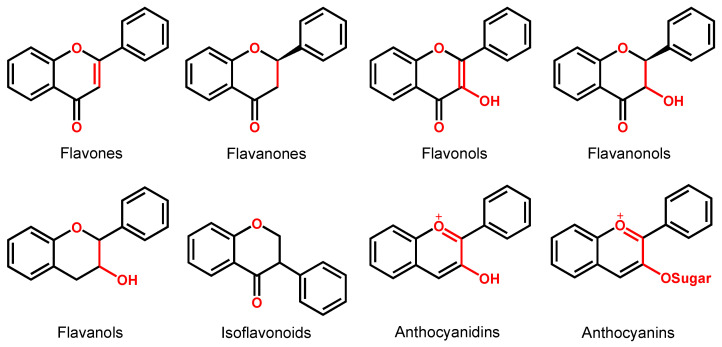
Chemical structures of the representative flavonoid groups.

Depending on their chemical structure, flavonoids can be classified into seven major groups (Figure 2): flavones (flavonones), flavanones, flavonols (flavon-3-ols), flavanonols (3-hydroxyflavanones), flavanols (flavan-3-ols), isoflavonoids (that correspond to an isomeric change on the B-ring bond), anthocyanidins and anthocyanins. More than 6000 flavonoids have been isolated and studied, with some of the most important being depicted in Table 1, along with the major sources of dietary intake.

The best described property of flavonoids is their antioxidant capacity. An antioxidant is a compound capable of retarding, delaying or inhibiting the oxidation of a substrate. Depending on their mechanism, antioxidants can be divided into: (i) primary antioxidants that function as free radical terminators (scavengers), (ii) secondary antioxidants that function by retarding chain initiation, and (iii) tertiary antioxidants that are related to the repair of damaged biomolecules. Flavonoid antioxidants are important ROS and RNS scavengers. The antioxidant capacity of flavonoids depends on their chemical structure, the number, position and redox properties of the phenolic –OH groups, and the structural relationship between the different substituents. Flavonoids’ in-vitro antioxidant capacity is well-established, being closely related to their redox behavior and electron-donor abilities. Moreover, though polyphenols are commonly known for their antioxidant effects, within certain physiological conditions, they can also exert a pro-oxidant action, producing free radicals that may be essential for the flavonoids’ ability to work as antimicrobial and antipathogenic therapeutic agents.

Electrochemical methods, such as cyclic voltammetry (CV), differential pulse voltammetry (DPV), square wave voltammetry (SWV), amperometry (A), have been widely used to study the redox properties of flavonoids, providing information about electron-transfer reactions and their redox potentials. Moreover, electroanalytical techniques have proved to be suitable for studying the total antioxidant capacity of flavonoids and other polyphenols and to achieve their determination in highly complex matrixes, such as fruits, vegetables, beverages, food supplements, pharmaceutical drugs and biological fluids [2,4,5,6,7]. Numerous electrochemical sensing strategies for the analysis of flavonoids have been developed based on functional electrodes and nanostructured materials [8,9], such as metal and carbon nanoparticles (NPs), nanorods (NRs), nanofibers (NFs), nanowires (NWs), carbon nanotubes (CNTs), graphene (Gr), graphene oxide (GO), reduced graphene oxide (rGO), quantum dots (QDs), metal–organic frameworks (MOFs), molecularly imprinted polymers (MIPs) [10] and other polymers, aerogels, DNA, and dendrimers (Den).

Understanding the flavonoid redox behavior that is intimately related to their antioxidant activity is essential for the development of new in-vitro methods of detection and quantification of flavonoids in food samples, supplements and nutripharmaceuticals. The application of the electrochemical methods in the routine quality control of natural products and foods, where the polyphenols’ antioxidant activity needs to be quantified in vitro, is of utmost importance. In this context, the objective of this review is to provide a comprehensive overview of the fundamental principles concerning flavonoid electron-transfer reactions, taking into consideration their antioxidant mode of action. The oxidation behavior of selected flavonoids will be described, and the phenol moiety oxidation pathways and the effect of substituents and experimental conditions on the flavonoids’ electrochemical behavior will be discussed. The second part of the review concerns the electroanalysis of flavonoids in natural products and their identification in highly complex matrixes, such as fruits, vegetables, beverages, food supplements, pharmaceutical compounds and human biological fluids, relevant for food quality control, nutrition, and health research.

## 2. Electrochemical Behavior of Flavonoids

Flavonoid redox behavior has been investigated mostly in aqueous and hydroalcoholic [2,11], but also in aprotic media [12], and a relationship between their electron-transfer properties and radical-scavenging activity has been found. In general, the antioxidant properties of phenolic compounds are conferred by the redox-active phenol groups. Thus, as with all polyphenols, the flavonoids’ voltammetric profile is directly related to their phenol content.

**Figure 3:**
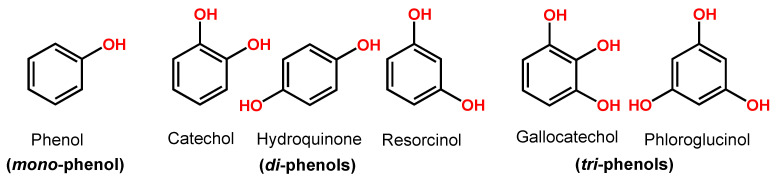
Phenol chemical structures.

Phenol is a compound with one hydroxy (–OH) group linked directly to a benzene ring. Catechol, hydroquinone and resorcinol (benzenediols) have two –OH groups, and gallocatechol and phloroglucinol (benzenetriols) have three –OH groups (Figure 3).

**Figure 4:**
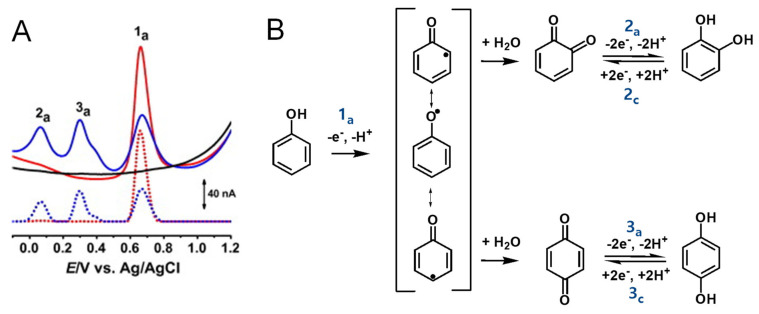
(**A**) Differential pulse voltammograms (DPVs) at GCE in pH 7.0 (▬) supporting electrolyte and 25 μM phenol: (▬) first and (▬) second scans, and DPVs baseline-corrected, (•••) first and (•••) second scans, and (**B**) phenol oxidation mechanism. Adapted from [13] with permission.

Phenol undergoes an irreversible, pH-dependent oxidation at the glassy carbon electrode (GCE), which occurs in one-step at the –OH group, with the transfer of one electron and one proton, corresponding to peak 1a at *E*_p_ = +0.65 V at pH = 7.0, vs. Ag/AgCl (3 M KCl) (Figure 4A) [13,14,15]. Phenol oxidation leads to the formation of a thermodynamically unstable phenoxy radical that coexists in three resonant forms (at the ortho- and para-positions with higher spin density, and at the meta-position with lower spin density), being immediately stabilized by hydrolysis, resulting in the formation of two electroactive oxidation products: ortho-quinone and para-quinone (Figure 4B). The ortho-quinone and para-quinone are further reversibly reduced—the ortho-quinone to catechol (peak 3c) and the para-quinone to hydroquinone (peak 2c) (Figure 4B)—in two parallel pH-dependent processes occurring with the transfer of two electrons and two protons each. The reversible peak potentials correspond to the catechol and hydroquinone electron-transfer mechanisms.

The electrochemical polymerization of phenol and its derivatives is known to produce thin, hydrophobic and insulating films at the electrode surface [16,17].

**Figure 5:**
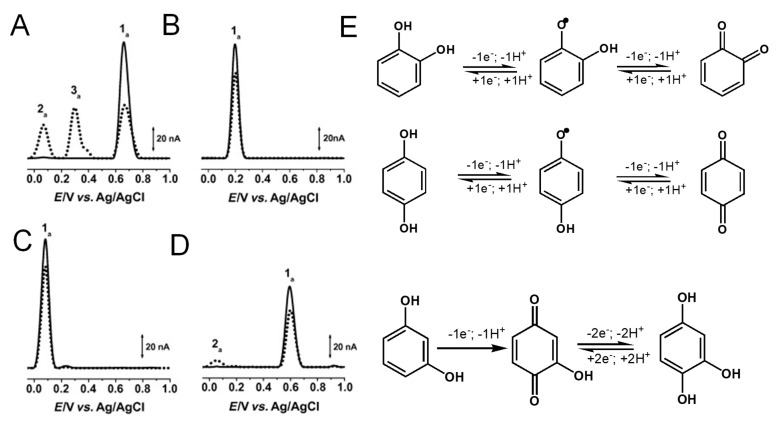
(**A**–**D**) DPVs baseline corrected at GCE, (▬) first and (▪▪▪) second scans, obtained in solutions at pH =7.0 of: (**A**) 25 μM phenol, (**B**) 25 μM catechol, (**C**) 25 μM hydroquinone, and (**D**) 25 μM resorcinol, and (**E**) oxidation mechanisms of catechol, hydroquinone and resorcinol. Adapted from [13] with permission.

Catechol and hydroquinone, with two –OH electron-donating groups, each follow one reversible pH-dependent oxidation at GCE, which occurs with the transfer of two electrons and two protons, corresponding to peak 1a at *E*_p_ ~ +0.20 V for catechol (Figure 5B) and at *E*_p_ ~ +0.08 V for hydroquinone (Figure 5C), at pH = 7.0, vs. Ag/AgCl (3 M KCl) [13,18,19,20]. The electrochemical reversible reactions and low oxidation potentials of catechol and hydroquinone moieties explain the higher in-vitro antioxidant capacity exhibited by flavonoids in which this phenol patterns is very common.

The di-phenol resorcinol meta-substitution does not allow for stabilization by resonance of the electrogenerated phenoxy radical; thus, its oxidation behavior is similar to that of a mono-phenol. Resorcinol undergoes a pH-dependent irreversible oxidation that takes place at a high positive potential—*E*_p_ ~ 0.60 V at pH = 7.0, vs. Ag/AgCl (3 M KCl)—with the transfer of one electron and one proton, and results in the formation of a catechol electroactive product [13,21,22] (Figure 5D).

Gallocatechol, with three –OH electron-donating groups, presents a reversible oxidation at GCE that occurs at low potential values, showing only small differences from catechol (Figure 5B) [11,23].

Flavonoids generally possess several phenolic –OH groups that determine their redox behavior. Thus, flavonoids present a common redox mechanism, associated with the electroactive –OH groups, but their oxidation is also influenced by the non-electroactive chemical substituents linked to the aromatic rings and by the experimental conditions, such as pH, applied potential, electrode material, electrolyte and solvent solutions, among other factors.

Many flavonoids have been characterized electrochemically, and the most representative are described in Table 2.

### 2.1. Electrochemical Behavior of Flavones, Flavonols, Flavanones, Flavanonols and Isoflavonoids

Flavones, flavanones, flavonols and flavanonols are all C4-keto flavonoids (Figure 2 and Table 1).

Flavones are among the most important subgroups of flavonoids and present a double bond between the carbons at the 2 and 3 positions and a ketone in position 4 of the C ring (Table 1). Like most flavonoids, they occur in edible plants and foods as β-glycosides, i.e., bound to one or more sugar molecules. They are known for their numerous biological activities, e.g., antioxidant, anti-inflammatory, anti-allergic, antifungal, hepatoprotective, antithrombotic, antiviral, and anticarcinogenic. Celery, olives, onion, lettuce, parsley, oregano, rosemary, thyme, green pepper, red wine, milk, chamomile tea, olive oil, peppermint oil, and *Ginkgo biloba* are some major sources of flavones.

**Figure 6:**
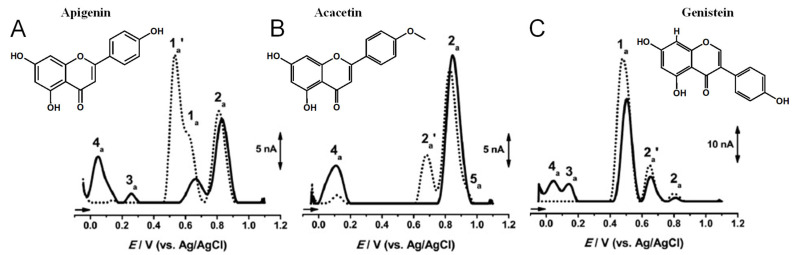
DPVs baseline corrected at GCE, (▪▪▪) first and (▬) second scans, obtained in a solution at pH = 7.0, of 10 μM: (**A**) apigenin, (**B**) acacetin, and (**C**) genistein. Adapted from [25] with permission.

Apigenin is a flavone that contains three –OH groups: one at position 4′ in the B ring, corresponding to a phenol structure, and two in the A ring at positions 5 and 7, corresponding to a resorcinol structure [25] (Table 1 and Figure 6). The flavone acacetin contains two –OH groups in the A ring at positions 5 and 7 that form a resorcinol-type structure (Figure 6). Apigenin and acacetin both undergo oxidations at GCE, with their processes being pH-dependent [24,25,26,27]. The first oxidation peak of apigenin is due to the irreversible oxidation of the –OH group at the 3′ position in ring B, occurring with the transfer of one electron and one proton. This peak is lacking in the case of acacetin, which does not possess the respective –OH group. In both compounds, the –OH groups forming the resorcinol structure in ring A are oxidized at a higher potential than ring B, and the formation of two redox products that are reversibly oxidized was observed.

The flavone luteolin contains four –OH groups: two at positions 3′ and 4′ in the B ring, forming a catechol structure, and two in the A ring at positions 5 and 7, forming a resorcinol structure (Table 1). Luteolin first undergoes an adsorption-controlled oxidation, which occurs with the transfer of two electrons and two protons, involving the oxidation of 4′–OH and 3′–OH moieties in ring B [28,29,30,31,63]. As in the case of apigenin and acacetin, the –OH groups of the resorcinol structure in ring A are oxidized at higher potential values.

Flavanones lack the double bond between carbons 2 and 3 in the C-ring of the flavonoid skeleton, which is present in flavones and flavonols (Table 1). They present important antiallergenic, antioxidant, antimicrobial, antihypotensive, vasodilatory and chemoprotective properties. They also show protective effects against atherosclerosis and cancer and reduce blood cholesterol and triglycerides. Flavanones are found in tomatoes and aromatic plants such as mint, but their major sources are citrus fruits, especially grapefruit. In fruits, flavanones exist as aglycons and glycosides; the main aglycones are naringenin in grapefruit, hesperidin in oranges, and eriodictyol in lemons.

Hesperidin is a major flavanone present in citrus fruits such as lemon, oranges, and satsuma mandarin. Its radical-scavenging and antioxidant properties are attributed to its chemical structure involving the electron-donating –OH group at position 3′ and the methoxy moiety of the B ring, and the –OH group at position 5 in the A ring. The electrochemical oxidation of hesperidin at the boron-doped diamond (BDD) electrode is an adsorption-controlled, irreversible process that occurs with the transfer of two electrons and two protons, leading to the formation of ortho-benzoquinone [64].

Flavonols have an unsaturated C ring at the carbons at positions 2 and 3 that is hydroxylated at carbon 3 and oxidized at carbon 4. Tea, red wine, fruits, and vegetables all represent important sources of flavonols. Again, the presence of phenolic –OH groups is responsible for their biological activities, particularly antioxidant activity. They also present anti-inflammatory, antiviral, anticarcinogenic, anti-asthmatic, cytoprotective, cardioprotective, hepatoprotective, nephroprotective, neuroprotective, and vasoprotective activities.

**Figure 7:**
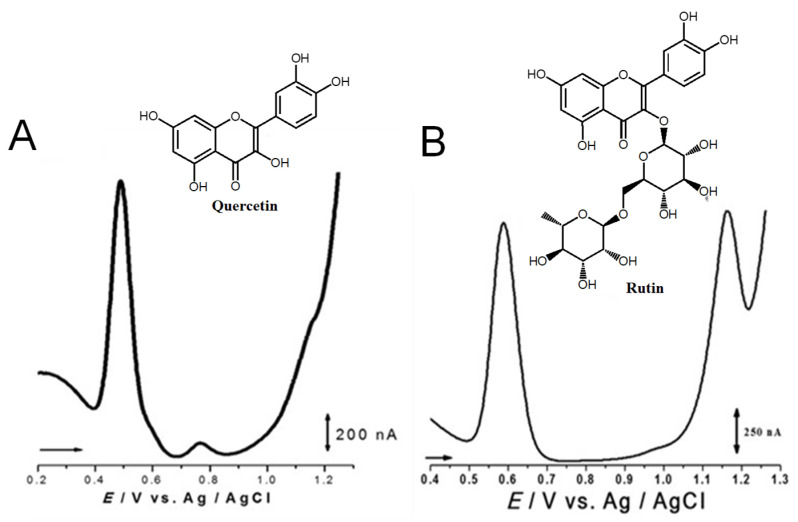
DPVs at GCE obtained in solutions of (**A**) 10 quercetin and (**B**) 100 μM rutin, at pH = 1.5. Adapted from [41,46] with permission.

Quercetin belongs to the flavonol subclass of flavonoids and is one of the major dietary antioxidants commonly present in onions, apples, kale, tea, and wine, as well as in numerous food supplements. In quercetin, of the five –OH groups present, the 3′ and 4′–OH electron-donating (catechol) groups of the B ring are the most easily oxidized, with their pH-dependent reversible oxidation occurring at very low positive potentials (related to the transfer of two electrons and two-protons), to produce an ortho-quinone product (Figure 7 and Table 1) [65]. The –OH group at position 3 in ring C is oxidized next, and it undergoes an irreversible oxidation reaction, also forming intermolecular hydrogen bonds with the neighboring oxygen. The other two –OH groups at positions 5 and 7 of ring A also have an electron-donating effect, and their oxidation is reversible [41]. The electrochemical behavior of quercetin was compared with that of kaempferol [66,67].

The flavonol rutin is a flavonoid glycoside with numerous pharmacological, nutraceutical, and chemotherapeutic properties. It exhibits high radical-scavenging and metal-chelating activities, but its low water solubility, stability, and possible interactions with other compounds limit its application in functional foods, food supplements, or drugs. The process of oxidation of rutin proceeds by a cascade mechanism that first involves the oxidation of the –OH groups at the 3′ and 4′ positions of the catechol group of ring B, in a reversible two-electron and two-proton transfer process (Figure 7 and Table 1). Next, the –OH groups at positions 5 and 7 of the resorcinol of ring A are irreversibly oxidized. Both mechanisms are highly pH-dependent and are either adsorption-controlled, diffusion-controlled or a combination of both [46,68].

Flavanonols are a class of flavonoids that have a 3-hydroxy-2,3-dihydro-2-phenylchromen-4-one backbone (Table 1). The most representative flavanonol is taxifolin that presents important anti-inflammatory activity and has the ability to inhibit the cholesterol synthesis and to prevent the synthesis and secretion of triacylglycerols and phospholipids. Taxifolin can be found in onion, milk thistle, Douglas fir bark, and French maritime pine bark, also being used for the preparation of several commercial products and pharmaceutical compounds. Taxifolin radical-scavenging activity was related to the presence of –OH groups at the 3′ and 4′ positions in ring B, at the 5 and 7 positions in ring A and at the 3 position in ring C. The oxidation of taxifolin at GCE in acetonitrile was studied, and its oxidation products were identified. The two-electron oxidation mechanism differs from that of flavonols (e.g., quercetin) due to the absence of the double bond between the carbons at the 2 and 3 positions [69].

Isoflavonoids are also C4-keto flavonoids, differing from the flavones, flavanones, flavonols and flavanonols isomers, by the position in which the B-ring is linked to the C-ring—at the carbon 3 position instead of the carbon 2 position (Figure 2 and Table 1). The antioxidant properties and redox behavior of isoflavonoids have been extensively studied. Due to their hormonal activity, they act as phytoestrogens in mammals, but they also exhibit pro-oxidant activity, leading to the generation of free radicals.

Genistein is an isoflavone that contains three –OH groups, one at position 4′ in the B ring and two in the A ring at positions 5 and 7, forming a resorcinol-type structure (Figure 6). Genistein undergoes irreversible, pH-dependent oxidation at the GCE, which occurs with the transfer of one electron and one proton from each –OH group and the formation of two electroactive oxidation products that further undergo two-electron and two-proton reversible redox reactions [5,26].

Biochanin is similar to genistein but the –OH group in the B ring is substituted by a methoxy group. Daidzein is also an isoflavone [70] that presents two –OH groups: one at position 4′ in the B ring and one at position 7 in the A ring. Both undergo two consecutive, irreversible, pH-dependent redox reactions, with the peak potential being influenced by the position of the –OH groups [26]. Daidzein can also be reduced to its end metabolite *S*-equol, a 7-hydroxy-3-(4′-hydroxyphenyl)-chroman, which presents a chemical structure close to the mammal hormone estradiol and has shown beneficial effects on the reduction of menopausal symptoms, as well as on the incidence of prostate cancer.

The irreversible oxidation of puerarin, the 8-*C*-glucoside of daidzein, using a nano-CeO_2_ CNT-modified GCE, was also investigated [71].

### 2.2. Electrochemical Behavior of Flavanols

Flavanols, also known as flavan-3-ols or catechins are derivatives of flavans and do not present a double bond between the carbons at the 2 and 3 positions, or a carbonyl in the C ring at the position 4. Catechin, epicatechin, epigallocatechin, gallocatechin, and their gallate derivatives are the most representative flavanols (Figure 2 and Table 1). They occur abundantly in tea leaves, cocoa beans, grape seeds, and fruits like grapes, apples, apricots and different types of berries. They exert powerful antioxidant activities and influence the molecular mechanisms involved in angiogenesis, extracellular matrix degradation, regulation of cell death, and multidrug resistance in cancers and age-related disorders.

**Figure 8:**
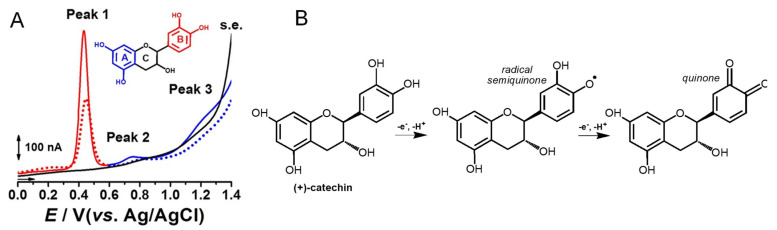
(**A**) DPVs at GCE, obtained in a solution of 10 μM catechin, at pH = 2.2: (▬, ▬) first scan, (•••, •••) second scan, and in (▬) supporting electrolyte (s.e.), and (**B**) proposed catechin oxidation mechanism. Reproduced from [72] with permission.

Catechin is a flavanol that presents two pharmacophores: two –OH groups that form a resorcinol moiety at the A ring, and two –OH groups that form a catechol moiety at the B ring (Table 1). It is generally found in fruits, cocoa products, green tea and wine. Catechin undergoes a pH-dependent oxidation at GCE, that proceeds in sequential steps, being related to the oxidation of the –OH groups of the catechol and resorcinol [59]. The reversible oxidation of the catechol 3′ and 4′–OH electron-donating groups occur first, at very low positive potentials (Figure 8). The 5 and 7–OH groups of the resorcinol moiety are oxidized next, in an irreversible oxidation reaction. Catechin presents strong adsorption onto the GCE, and its final oxidation product is not electroactive and blocks the electrode surface. The knowledge concerning the catechin electrochemical behavior enabled us to establish the relationship between its voltammetric behavior and its strong in-vitro antioxidant capacity [72].

The ortho-methylation of catechol groups in catechin derivatives was also investigated [43], and their antioxidant capacity is lower compared to the non-methylated parent compound when the reaction is carried out at the catechol on the B-ring.

### 2.3. Electrochemical Behavior of Anthocyanins and Anthocyanidins

Anthocyanins are glycosides composed of the anthocyanidin aglycone with one or more glycosidically bonded mono- or oligosaccharidic units. The anthocyanidins (or aglycons) consist of an aromatic A ring bonded to a heterocyclic C ring that contains oxygen, which is also bonded by a C–C bond to a third aromatic B ring. Although both anthocyanins and anthocyanidins lack the keto group at the C-ring (Table 1), they have always been included in the flavonoids class. They are natural plant pigments with a red, blue, or purple color, presenting beneficial effects for the plant and for human and animal health. Dietary sources of anthocyanins and anthocyanidins include berries and red-skinned grapes, apples, and pears, and various vegetables such as radishes and red/purple cabbage, black rice and black beans.

A series of anthocyanins and anthocyanidins were characterized electrochemically (Table 2), with their redox potentials being low, in agreement with their good radical-scavenging activities [73,74]. As in the case of other flavonoids, the higher the number of phenolic –OH groups in the anthocyanins/anthocyanidins structure, the greater their radical-scavenging activity [62]. The antioxidant activity of anthocyanins and anthocyanidins is strongly influenced by pH, being optimum in the 5.0 < pH < 7.4 range.

In anthocyanins and anthocyanidins, the steric hindrance caused by the introduction of large sugar moieties, especially when near to electroactive –OH electroactive groups, leads to a positive anodic shift, whereas the electron-donor character of methyl substituents leads to a negative anodic shift, consistent with an easier oxidation. On the other hand, the methoxylation of the electroactive phenolic groups can lead to the suppression of the anodic processes. For this reason, the –OH group of the anthocyanin myrtillin chloride at the 4′ position from pyrogallol on the B-ring is more easily oxidized than the –OH group in the ortho-position to two methoxy groups in oenin chloride, which is also easier to oxidize than the catechol group in the ortho-position to a methoxy group in petunidin chloride. A reversible oxidation reaction occurs only with a methoxy group in the ortho-position to the –OH group at the 4′ position. Electrochemical studies showed an irreversible reaction corresponding to the oxidation of the –OH group at the 4′ position of the B-ring for the compounds in which this group is located in the ortho-position to two methoxy groups, such as in malvin and oenin chloride. –OH groups from the B-ring do not present variation in their oxidation peak potential values due to the effect of glucosylation on the A-ring. At higher positive potentials, an irreversible oxidation reaction was observed, corresponding to the oxidation of the –OH groups of the A-ring.

### 2.4. Comparison of Flavonoid Electrochemical Behavior and Antioxidant Activity

In general, flavonoid antioxidant activity is highly correlated with the first electrochemical oxidation potential, as the redox-active phenol moiety common to all flavonoids is what confers their antioxidant properties. [2,37,75,76,77,78]. The most effective superoxide radical quenchers are the flavonoids with either a pyrogallol moiety at any position of the flavonoidal nucleus or a catechol (ortho-dihydroxy) group on the B ring along with an –OH group at the 3 position on the C ring [77]. Medium superoxide-scavenging activity was observed for polyphenolic flavonoids that lack any of the abovementioned structural features, while the lowest superoxide-scavenging activity was observed for monophenolic flavonoids.

Depending on the chemical structure, flavonoids are oxidized in one, two or three steps, corresponding to the electroactive moieties: catechol/gallate, phenol and resorcinol. The phenolic groups on ring B and ring A have a greater antioxidant activity when compared to ring C. Flavonoids’ electrochemical oxidation processes are frequently coupled with homogenous chemical reactions. Flavonoids’ oxidation products are generally associated with electroactive adsorbed films with reversible electrochemical behavior that, after successive voltammograms, result in electrode passivation. Moreover, the low peak potentials observed for these oxidation products and the reversibility of their anodic oxidation are in agreement with the presence of catechol-/hydroquinone-like moieties in flavonoid oxidation products.

Flavonoid –OH groups in para- and ortho-positions are oxidized at lower potentials than the –OH groups in the meta-position. Also, the mono-substituted –OH group on the A-ring requires a higher potential to be oxidized than the –OH group on the B-ring [35,37]. Therefore, it is well-established that the electron-donor ability of flavonoids is higher in the flavonoids presenting a catechol moiety on B-ring, such as quercetin, quercitrin, rutin, luteolin, morin, etc. Thus, since the deprotonation is easier, the resorcinol moiety on the B-ring oxidizes at a close but slightly lower potential when compared with the same group at the A-ring. This relevant effect is illustrated by the oxidation behavior of morin, a tetrahydroxy flavonoid that occurs with the transfer of one electron and one proton.

The importance of the –OH groups’ positions on the electron-transfer mechanism was illustrated by correlating the oxidation peak potentials of the flavone apigenin and its isomer genistein, which differ only by the position of ring B relative to ring C [26]. Although their oxidation mechanisms are similar, the structural difference influences their redox behavior. The lower oxidation potential of genistein (and higher antioxidant activity) in comparison to apigenin is due to the influence of the O atom at position 4 in ring C. In the case of genistein, the electronegativity of the O atom in ring C partially displaces the delocalized electron cloud in ring B, facilitating the oxidation. Contrarily, a higher distance between the oxygen atom in ring C and the –OH group in ring B of apigenin is correlated with a higher oxidation potential value.

Flavonols are normally oxidized at lower potentials when compared with flavones since the hydrophilic substituents improve the solubility, diffusion, and proton-donor ability in aqueous media [75,79]. Moreover, it has been shown that the diastereomization has little effect on flavonoid electrochemical oxidation, consistent with the catechin and epicatechin similar electrochemical behavior and comparable antioxidant capacity [37]. The –OH group at the 3 position in flavonoids might improve their hydrosolubility, enhancing the electron-transfer reactions, while the double bond between carbon 2 and 3 might contribute to the electron delocalization, allowing the stabilization of the phenoxy radical and decreasing the oxidation peak potential. This explains the slight anodic shift and low oxidation peak potential observed for morin [52] and kaempferol [80], both lacking the catechol moiety on the B ring. The C4 keto group may exert a dual function on flavonoid antioxidant capacity, with the –OH group at 3 and/or 5 positions enabling a bidentate metal chelation site, which has a preventive influence on the Fenton oxidative mediated processes. The C4 keto group decreases the electron density of conjugated –OH groups, enhancing their acidity and reducing their electron-donor ability [81,82].

As observed for anthocyanins and anthocyanidins, the flavonoid steric hindrance effect on the electron-transfer reactions due to the presence of sugar moieties, particularly near electroactive –OH electroactive groups, leads to a positive anodic shift, whereas the electron-donor character of methyl substituents leads to a negative anodic shift, consistent with easier oxidation and increased antioxidant activity.

Due to the fact that flavonoid oxidation potentials are sensitive to the arrangement of –OH groups, a discrepancy between the scavenging activity and the oxidation potential of polyphenolic flavonoids that possess zero (e.g., galangin and 7-hydroxyflavonol) or one –OH group (e.g., 4′,7-dihydroxyflavone, apigenin, genistein) on the B ring was observed [77]. In fact, these flavonoids, although they present high oxidation potentials, have EC_60_ values (expressed as the concentration of flavonoid needed to consume 40% of a superoxide radical) similar to flavonoids with low oxidation potentials like luteolin and rutin. Except these five flavonoids, a high correlation between EC_60_ values and electrochemical oxidation potentials was observed, indicating that the superoxide radical-scavenging activity of flavonoids is mainly governed by their susceptibility to oxidation [77].

Flavonoid antioxidant activity increases with the concentration of flavonoids reaching a maximum where pro-oxidative behavior occurs [83]. The pro-oxidant potency of flavonoids is dependent on the presence of atmospheric oxygen and follows the order quercetin > rutin > epigallocatechin gallate > catechin. Moreover, the pro-oxidant activity is typically catalyzed by metals, particularly transition metals such as Fe and Cu, present in biological systems [84].

## 3. Electroanalytical Determination of Flavonoids in Natural Samples

Electrochemical sensing methods have been extensively used for the direct, sensitive and selective determination of flavonoids in complex matrixes such as food, beverages, pharmaceuticals, and biological samples. In natural samples, generally, different flavonoid species coexist, each possessing different numbers and positions of phenolic –OH groups and non-electroactive substituents; thus, flavonoid qualitative determination using electrochemical methods is based on the identification of each electroactive compound.

Examples of electrochemical quantitative analysis of some of the most representative flavonoids present in biological matrixes, as well as in food and folk and/or traditional medicines, are shown in Table 3 and Table 4.

### 3.1. Flavonoid Determination in Plant-Based Samples

Flavonoids are known to be ubiquitous in foods, such as fruits, vegetables, legumes, spices, and medicinal plants. However, in plant matrixes, flavonoids may occur in free forms (aglycones), as glycosylated or acylated derivatives, and as oligomeric and polymerized structures, such as the flavan-3-ol-derived condensed tannins (also called proanthocyanidins). They may also be found linked to plant matrix components like cell walls, carbohydrates or proteins. Flavonoids’ structural diversity and the complexity of the plant matrix affect their electroanalytical determination [9], and sample preparation and flavonoid extraction methods are crucial [214]. Many studies focused on the development of extraction and purification methods of flavonoids from [215]: (i) pulp (the extraction depends on the wax content and the flesh density), (ii) flowers (easier extraction that depends on the presence of coloring compounds, essential oils, terpenes, carotenoids, organic acids, mucilages and waxes), (iii) peels (the extraction depends on the presence of volatile oils, pectin, natural pigments and dietary fiber), (iv) seeds and seed oils (the extraction is influenced by the high content of fatty acids and essential oils), (v) leaves (the extraction depends on the chlorophyll and, in thick and fleshy leaves, on the presence of waxes and resins), (vi) barks of some species of pines and other trees (flavonoid recovery is difficult due to the content of cellulose, hemicellulose, lignin, and fatty compounds associated to the cell walls), (vii) roots (the extraction depends on the presence of resins and essential oils), (viii) stems (the extraction depends on the presence of lignins and polysaccharides), and (ix) grains (the extraction is difficult due to the complex chemical constitution; factors like humidity and temperature during grain storage can change flavonoid properties). Therefore, although many studies reported the determination of flavonoids in natural plant and food samples, no standardized procedures for the determination of all flavonoid groups were reported.

#### 3.1.1. Flavonoids Determination in Food

The flavone luteolin was electrochemically detected in red wine and peach juice using a sensor consisting of a GCE modified by chitosan (CS) and rGO aerogel combined with dispersed ZrO_2_ NPs, which showed a limit of detection (LOD) of 1 nM in the linear range from 5 nM to 1000 nM [130]. The luteolin determination in grape juice was obtained using a nanocomposite-based sensor, formed by Ti_3_C_2_-MXene highly porous zeolite imidazolate frameworks (ZIF) and CNTs immobilized onto GCE, which allowed for an LOD of 0.03 nM in the linear range from 0.1 to 1000 nM [131]. Luteolin in green tea was detected at a zirconium-fumarate MOF and mesoporous carbon (MC) nanocomposite, with an LOD of 2.9 nM in the linear ranges from 0.02 to 0.2 μM and from 0.2 to 10 μM [121].

The quantification of the flavanone hesperidin in dried tangerine peel samples at a GCE modified by MIPs deposited on an ultrafine electro-polymerized activated carbon-Au NPs nanocomposite was also reported [110]. The complex sensor design allowed for an LOD of 45 nM in the linear range from 85 nM to 30 μM. Hesperidin detection in samples of fortified fruit juice was achieved at a nano-graphene-platelet/Brilliant-green (nGp-Bg) composite-modified CPE, with an LOD of 50 nM in the linear ranges from 0.1 to 7 μM and from 7 to 100 μM [114]. Hesperidin determination in orange juice and in laboratory samples was also reported at a multiwalled carbon nanotubes (MWCNTs)-modified basal-plane pyrolytic graphite electrode (BPPGE) and at screen-printed electrode (SPE), showing an LOD of 0.61 μM for CV and 7 nM for adsorption stripping voltammetry (AdSV), in a linear range up to 30 μM [113].

The flavonol quercetin was voltametrically determined in green apples using a GCE modified by Ag NPs anchored onto a porous ultrathin graphitic carbon nitride (gCN) nanosheet, which led to an LOD of 6 nM in the linear range from 0.1 nM to 0.12 mM [163]. The quantification of quercetin-3-glucoside, quercetin-4′-glucoside and quercetin-3,4′-diglucoside in apple peel, onion, and tartary buckwheat was also performed at a long-length CNT electrode [216].

The flavonol rutin was determined in buckwheat seeds using an ionic liquid (IL) 1-hexyl-3-methylimidazolium-bis(trifluoromethylsulfonyl)imide-modified carbon paste electrode (CPE), with the sensor showing an LOD of less than 5 nM in the linear range from 5 to 80 nM [181]. Rutin was also quantified in samples of red apple, red onion, oat, orange, strawberry and salvia, using a poly(sulfosalicylic acid) (PSSA) film electropolymerized onto an Au electrode modified by a self-assembled monolayer (SAM) of 2-mercaptobenzothiazole (MBT) and MWCNTs. The PSSA/MWCNTs/MBT/Au sensor showed an LOD of 1.8 nM in the linear ranges from 0.01 to 0.8 μM and from 0.8 to 10 μM [182]. In another report, a magnetic nanocomposite prepared from electrospun CoFe_2_O_4_ NFs and GO was used to develop a sensor for the detection of rutin in red apple, lime, lemon and orange juices, which presented an LOD of 0.94 pM in the linear ranges from 0.01 to 0.8 μM and from 0.8 to 10 μM [183]. The detection of rutin in oranges, pharmaceutical tablets, and human serum was also achieved at a N and S co-doped carbon dots (N, S@C-dots)-modified GCE, with an LOD of 0.8 nM in the linear range from 2 to 1300 nM [186].

The quantification of the flavonol quercetin in apple and pear juices and red and green tea was detected at a bare GCE, using a ratiometric electrochemical sensing methodology that achieved an LOD of 3.1 nM in the linear range from 0.1 to 15 μM [157]. Quercetin in apple juice was also detected at a cathodically pretreated BDD (CPT-BDD) electrode in a cetyltrimethylammoniumbromide (CTAB) media, with an LOD of 0.44 nM in the linear range from 1.7 nM to 0.33 μM. In a different report, the quercetin determination in different brands of tea samples was conducted at a GCE modified by a 3D hybrid material consisting of 7-propinyloxy-3-(p-propinyloxyphenyl) coumarin bearing double terminal ethynyl groups and bounded to SWCNTs, with an LOD of 20 nM in the linear range from 0.25 to 3 μM [166]. Using nanocomposites-based sensors, the quercetin determination was achieved: (i) in apple juice, at a carbon-modified kaolin clay-composite electrode that showed an LOD of 0.057 mM, in the linear range from 0.12 to 182.1 mM [155]; (ii) in grape, apple and pear juices, honey, green and black tea, as well as blood, human breast milk and urine, at a GCE modified by a lotus flower like SeO2-decorated rGO nanocomposite that showed an LOD of 1.6 nM, in the linear range from 0.0 to 200 μM [159]; (iii) in green apple, green tea and honeysuckle, at a GCE modified by graphitic carbon nitride/nickel oxide (g-C_3_N_4_/NiO) nanocomposites that showed an LOD of 2.0 nM in the linear range from 0.010 to 250 μM [160]; (iv) in tea and honeysuckle, at a GCE modified by a β-cyclodextrin/graphene nanocomposite that showed an LOD of 1.0 nM in the linear range from 0.005 to 20 μM [161]; and (v) in peanut hulls, at a GCE modified by Au QDs and Au NPs that showed an LOD of 2.0 nM in the linear range from 0.01 to 6.0 μM [165].

For the detection of the flavanonol taxifolin in peanut oils, an electrochemical sensor based on SPEs was proposed that allowed an LOD of 0.021 μM in the linear range from 0.05 to 1 μM [208].

The health benefits of consuming green tea, including the anti-inflammatory, antiarthritic, antibacterial, antiangiogenic, antioxidative, antiviral, and neuroprotective effects, and its role in preventing cancer and cardiovascular diseases, have been well documented. Many of the beneficial actions of green tea are related to its flavanol content, although the presence of other classes of flavonoids has also been reported. Several strategies were used for the detection of the flavanol catechin in green tea using electrochemical sensors: (i) a gallic acid/MWCNT/CPE sensor showed an LOD of 0.017 μM in the linear range from 0.10 to 2.69 μM [94]; (ii) a sensor based on laccase-Au NPs encapsulated-PAMAM Den bonded onto a 3′,4′-diamine-2,2′;5′,2′′-terthiophene (PDATT) conducting polymer showed an LOD of 0.05 μM in the linear range from 0.1 to 10 μM [95]; (iii) a sensor consisting on the SAM of an Ni(II) complex with 3-mercaptopropionic acid immobilized onto an Au electrode showed an LOD of 0.83 μM in the linear range from 3.31 to 25.3 μM [96]; (iv) a sensor consisting on triaminotriazine (TAT)-based polyimide (PI) films immobilized onto a Pt electrode showed an LOD of 15.2 μM in the linear range from 50 to 350 μM [97]; (v) a carboxylic group functionalized single-walled carbon nanotubes (SWCNTs) and poly(hydroxymethylated-3,4- ethylenedioxythiophene) (PEDOTM)-modified GCE showed an LOD of 0.013 μM in the linear range from 0.039 to 40.84 μM [98]; (vi) a sensor consisting on CNTs and carboxymethylcellulose (CMC) thin-film electrodes (CMC-CNT) showed an LOD of 0.06–0.12 μM in the linear range from 5 to 194 μM [99]; (vii) a sensor based on MIP-rGO and ZIF-8-modified GCE showed an LOD of 0.003 nM in the linear range from 0.01 nM to 10 μM [100]; (viii) a sensor based on nanocomposites of Pt NPs decorated on MnO_2_ and CNTs immobilized onto GCE showed an LOD of 0.02 μM in the linear range from 2 to 950 μM [101]; (ix) a poly-Methylene Blue (PMB)-modified CPE showed an LOD of 4.9 nM in the linear ranges from 0.1 to 1 μM and from 1 μM to 1.0 mM [102]; and (x) a sensor based on porous N-doped Gr-modified GCE showed an LOD of 0.088 μM in the linear range from 1.0 to 30 μM [103].

Catechin was also electrochemically detected in cocoa powder at a regenerable carbon black (CB) and MoS_2_ nanohybrid immobilized onto SPE; this methodology allowed for an LOD of 0.017 μM in the linear range from 0.12 to 25 μM [88]. In another report, catechin determination in bioactive plant extracts was achieved using carbon SPEs and batch injection analysis (BIA) [89]. Moreover, catechin in red wine was determined at a GCE modified by Pt and MnO_2_ at functionalized MWCNTs, with an LOD of 0.02 μM in the linear range from 2 to 950 μM [101].

#### 3.1.2. Flavonoids Determination in Medicinal Herbs

Medicinal herbs and pharmaceutical preparations obtained from plants can be important sources of indispensable elements and flavonoids for humans. Therefore, in recent years, flavonoid determination in herbal drugs and folk medicine remedies has received special attention.

**Figure 9:**
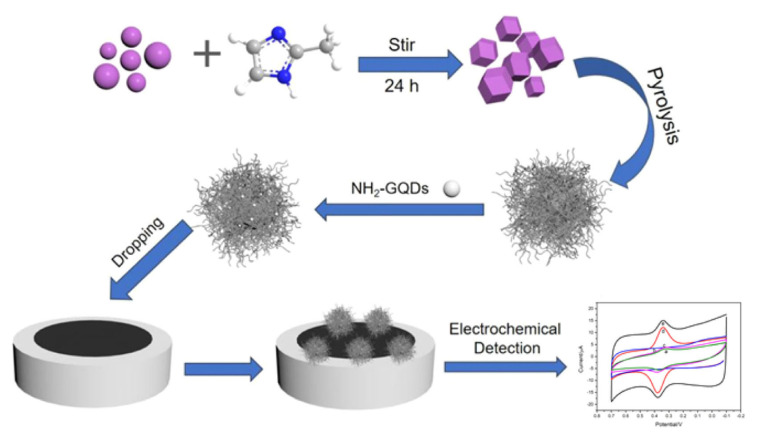
Schematic representation of an electrochemical sensor for the detection of luteolin, based on ZIF-derived Co_3_O_4_ at N-doped CNTs-amino-functionalized Gr QDs composites modified GCE (Co_3_O_4_@N-CNTs/NH_2_-GQD/GCE). Adapted from [129] with permission.

An example is the voltammetric determination of the flavone luteolin in real samples of *Chrysanthemum*, a flower with a well-known history in traditional Chinese and Korean medicine, used for the treatment of inflammation, hypertension and respiratory diseases. Different designs for the development of electrochemical sensors have been employed: (i) a GCE modified by MWCNTs and IL 1-butyl-3-methylimidazolium hexafluorophosphate (BMIMPF6) nanocomposite that showed an LOD of 0.5 nM in the linear range from 5 nM to 1 μM [122]; (ii) a GCE modified by MWCNTs and poly(crystal violet) (PCV) that showed an LOD of 5 nM in the linear range from 0.02 nM to 70 μM [123]; (iii) an Au/Pd/rGO nanofilm-modified GCE that showed an LOD of 0.98 nM in the linear range from 0.01 to 80.0 μM; (iv) a GCE modified by a MoO_3_, PEDOT and CD-MOF nanocomposite that showed an LOD of 0.1 nM in the linear range from 0.0004 to 1.8 μM [127]; (v) a GCE modified by a zirconium MOF UiO-66/rGO composite that showed the LOD of 0.75 nM in the linear range from 0.001 μM to 20 μM [128]; and (vi) a GCE modified by ZIF-derived cobalt trioxide at N-doped CNTs and NH_2_-functionalized Gr QDs nanocomposites that showed an LOD of 0.1 nM in the linear range from 0.5 to 1000 nM [129] (Figure 9).

A sensor based on Au NP and boron nitride nanosheet (BNNS)-modified GCE was employed for the determination of luteolin in Perilla (*Perilla frutescens* L.) leaves, which have shown therapeutic efficacy in the treatment of inflammatory disorders, allergies, and bronchial asthma. A LOD of 1.7 pM for luteolin, in the linear ranges from 5 to 1200 pM and from 0.02 to 10 μM, was achieved [63].

The flavone baicalein was electrochemically detected in the Chinese herb *Oroxylum indicum*, as well as in human urine samples, using a GCE modified by bismuth oxide-carboxylated MWCNTs that showed an LOD of 2.0 nM in the linear range from 0.01 to 15 μM [85]. Baicalein in the Chinese medicine *Scutellaria baicalensis Georgi* was quantified using a GCE modified with MoO_3_ and poly (3,4-ethylenedioxythiophene) (PEDOT) NWs nanocomposites, with the sensor showing an LOD of 1.5 nM in the linear range from 0.005 to 0.86 μM [86].

The flavonol quercetin quantification in different samples of tea and honeysuckle from dried flowers used as a medicine was achieved at a CPE modified with porous alumina microfibers (AM), which showed an LOD of 10 nM in the linear range from 0.025 to 1.5 μM [167].

Different methodologies were used to quantify the flavanonol taxifolin in *Polygoni Orientalis Fructus*, a clinically effective Chinese medicine, based on voltammetric sensors consisting of: (i) electrodeposited rGO (E-rGO) films grown in a preferential vertical orientation onto GCE (E-rGO/GCE), which showed an LOD of 2 nM in the linear range from 10 nM to 1.0 μM [209]; (ii) Pd NPs on poly(diallyl dimethyl ammonium chloride) (PDDA)-functionalized Gr composite immobilized onto GCE, which showed an LOD of 1 nM in the linear range from 40 nM to 1 μM [210]; (iii) Co_3_S_4_@MoS_2_ loaded onto immobilized GCE, which showed an LOD of 1.67 nM in the linear range from 5 nM to 1 μM [129]; (iv) MoS_2_ and N-doped active carbon composite immobilized onto GCE, which 211 an LOD of 0.3 nM in the linear range from 1 nM to 1 μM [212]; and (v) Ni-based MOF and CNTs composite electrode, which showed an LOD of 13 nM in the linear range from 40 nM to 10 μM [208].

### 3.2. Flavonoids Determination in Nutraceuticals

Many popular nutraceutical and dietary supplements that consists of mixtures of fruit and medicinal plant extracts containing flavonoids are expected to provide great antioxidant, antimicrobial, anti-inflammatory effects; therefore, quality control methodologies to achieve the analytical quantification of their flavonoid content are required.

The flavone luteolin was electrochemically detected in *Duyiwei* capsules at a carbon IL electrode (CILE), modified by a Pt NPs-decorated biomass porous carbon (BPC) nanocomposite. The sensor achieved LOD of 2.6 nM in the linear range from 0.008 to 100.0 μM [132]. In another strategy, luteolin in *Duyiwei* capsules was determined at a GCE modified by magnetic MIP, Fe_3_O_4_ and rGO, with an LOD of 3 pM, in the linear ranges from 0.01 to 1 nM and from 1 nM to 50 μM [133].

The electrochemical detection of the flavanone hesperidin in commercially available dietary supplements was achieved using an unmodified BDD electrode, which achieved an LOD of 1.2 μM in the linear range from 4.1 μM to 0.1 mM [64]. Hesperidin quantification in traditional Chinese medicines was also performed at a mesoporous SiO_2_-modified CPE, with an LOD of 0.25 μM, in the linear range from 0.5 to 25 μM [115]. In another report, hesperidin determination in similar samples was achieved with an LOD of 8.2 nM in the linear range from 0.005 to 8.0 μM, using an Au NPs/rGO/GCE sensor [116]. Hesperidin was also voltametrically detected in a pharmaceutical formulation at an electro-activated disposable pencil graphite electrode (ePGE), with an LOD of 0.2 μM in the linear range from 0.5 to 10 μM [117].

The flavonol fisetin was quantified in commercial nutritional supplement formulations using a cathodically pre-treated BDD (CPT-BDD) electrode. The method showed an LOD of 0.28 μM in the linear range from 1.7 to 6.9 μM [108].

The flavonol quercetin was determined in *Ginkgo* tablets using a sensor consisting of a Pt-Au-BPC nanocomposite, which showed an LOD of 50 nM in the linear ranges from 0.15 to 6.0 μM and from 10 to 25 μM [171]. Quercetin determination in different pharmaceutical samples was also conducted at a sensor based on magnetic rGO (MrGO), Fe_3_O_4_ and Ag NPs integrated into a MIP-modified SPE [169], with an LOD of 13 nM and a linear response range from 20 nM to 250 μM.

**Figure 10:**
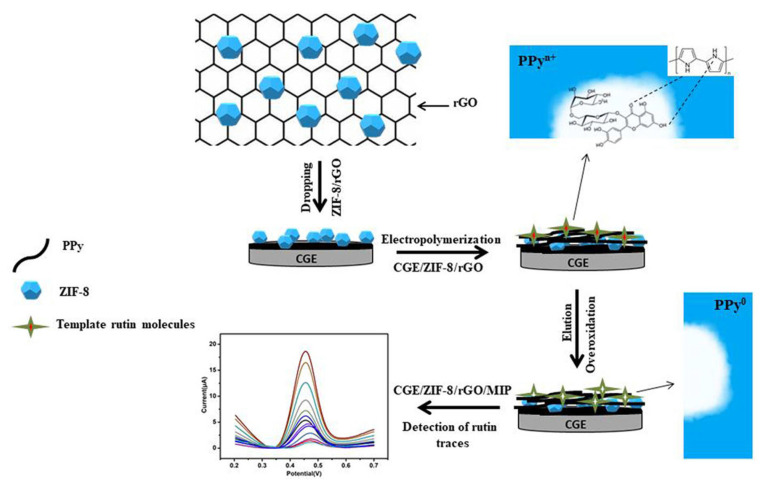
Schematic representation of an electrochemical sensor for highly recognition of rutin, based on MIP decorated onto a GCE-modified ZIF-8 and rGO surface. Adapted from [185] with permission.

Another example is the determination of rutin in tablets and pharmaceutical formulations. Different electrochemical sensors have been proposed, and examples may include: (i) a Gr and Au NPs-modified stainless steel acupuncture needle (AN) electrode that showed an LOD of 25 nM in the linear ranges from 0.08 to 10 μM and from 0.02 to 20 mM [187]; (ii) a GCE modified by Methylene Blue (MB)-loaded ZIF-8 crystals deposited onto rGO that showed an LOD of 20 nM in the linear range from 0.1 to 100 μM [188]; (iii) a GCE modified by Ni NPs nanoparticles incorporated with GO composite, which showed an LOD of 3.2 nm in the linear ranges from 0.01 nM to 1 μM and from 2.2 to 15 μM [193]; (iv) a GCE modified by MIP decorated onto a ZIF-8 and rGO composite that showed an LOD of 0.1 nM in the linear ranges from 0.0005 to 0.05 μM and from 0.05 to 100 μM [185] (Figure 10); (v) a CNTs paste electrode (CNTPE) that showed, in one report, an LOD of 50 nM in the linear ranges from 0.08 to 1.4 μM and from 2.0 to 160 μM [192], in a second report, an LOD of 34 nM in the linear range from 0.2 to 10 μM [190], and, in a third report, an LOD of 33.9 nM in the linear range from 48 to 960 μM [201]; (vi) a Cu(II)-resin carbon composite electrode that showed an LOD of 26.5 nM in the linear range from 1 to 8 μM [191]; (vii) a Gr-Au NPs SPCE that showed an LOD of 11 nM in the linear ranges from 0.01 to 1 μM and from 2.2 to 15 μM [194]; (viii) a porous carbon-encapsulated Mg-Al-Si alloy (Mg-Al-Si@PC) nanocluster-modified GCE that showed an LOD of 0.01 μM in the linear range from 1 to 10 mM [195]; (ix) a GCE modified by plated lead film (LF) that showed an LOD of 0.25 nM in the linear range from 0.5 to 10 nM [196]; (x) an N-doped Gr supported Au-Ag nanothorn (Au-AgNTs/NG) that showed an LOD of 15 nM in the linear range from 0.1 to 420 μM [197]; (xi) a single-sided heated graphite cylindrical (ss-HGC) electrode that showed an LOD of 1 nM in the linear range from 4 nM to 1 μM [198]; (xii) a black phosphorene (BP) nanosheet-modified GCE incorporated with poly (3,4–ethylenedioxythiophene) (PEDOT) and poly(styrenesulfonate) (PSS) that showed an LOD of 7 nM in the linear ranges from 0.02 to 15 μM and from 15.0 to 80 μM [199]; (xiii) a DNA-IL-CP electrode that showed an LOD of 1.3 nM in the linear range from 8 nM to 10 μM [200]; (xiv) a poly(p-aminobenzene sulfonic acid) (PABSA)-modified GCE that showed an LOD of 100 nM in the linear range from 0.25 to 10 μM [202]; (xv) a CB/WO_3_-modified carbon SPE that showed an LOD of 2 nM in the linear range from 0.01 to 75.5 μM [203]; (xvi) a CoWO_4_ nanosheet and porous carbon (PC) sensor that showed an LOD of 0.45 ng/mL in the linear range from 5 to 5000 ng/mL [204]; and (xvii) a MWCNTs and Al-based MOF CAU-1-modified GCE that showed an LOD of 0.67 nM in the linear range from 1.0 nM to 3.0 μM [205].

The flavonol kaempferol in *XinDaKang* tablets was detected at an electrochemical sensor based on GCE modified with a nonocomposite consisting of MOF MIL-100(Fe), MWCNTs and PEDOT, and it showed a linear range from 50 to 1950 nM and an LOD of 13.2 nM [118].

A sensor for the detection of the flavonol galangin in pharmaceutical formulations and human urine was also proposed [109], consisting of a GCE modified by a three-dimensional porous rGO and nafion (NAF) nanocomposite that achieved with an LOD of 1.11 nM in the linear range from 0.02 to 45 μM.

### 3.3. Flavonoids Determination in Human Biological Fluids

Aiming to understand the flavonoids’ mechanism of action and to develop new methodologies for controlling their adequate intake, several methodologies concerning the electrochemical quantification of flavonoids in human body fluids have been developed. However, flavonoids are absorbed and metabolized following ingestion; therefore, the determination of flavonoids in biological fluids, such as urine and blood (plasma or serum), where their concentrations are much reduced, is challenging. Therefore, most of the experiments concerning flavonoid detection in biological fluids were based on spike-and-recovery experiments of known flavonoid concentrations.

A sensor based on a GCE modified by a porphyrin-based zirconium MOF 525 and macroporous carbon (MPC) composite showed a low LOD of 0.35 nM in the linear ranges from 5 nM to 0.1 μM and from 0.1 to 5 μM, and a good ability for the detection of the flavone luteolin in human serum and urine [119]. Luteolin determination in human urine was also achieved using a GCE modified by Au nanoflakes (NFs)-decorated BPC nanocomposites, with an LOD of 0.07 μM in the linear ranges from 0.15 to 1.8 μM and from 1.8 to 10 μM [120].

**Figure 11:**
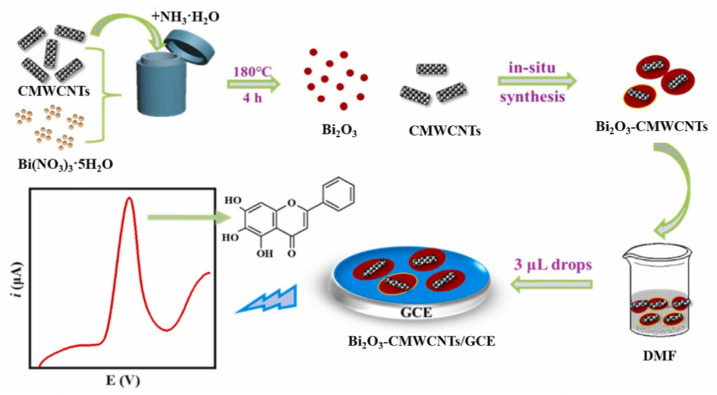
Schematic representation of an electrochemical sensor for the detection of baicalein, consisting on a GCE modified by bismuth oxide-carboxylated MWCNTs. Adapted from [85] with permission.

The detection of the flavone baicalein in human urine was achieved at a GCE modified by Bi_2_O_3_ and CMWCNTs, with an LOD of 2.0 nM in the linear range from 0.01 to 15 μM [85] (Figure 11).

A CuWO_4_ and polyaniline (PANI) nanocomposite-modified GCE was developed for the determination of quercetin in adult male blood and urine and pregnant female blood and urine samples, which showed an LOD of 1.2 nM and a wide linear range from 0.001 to 0.500 μM [174]. Quercetin in plasma was determined with an LOD of 7 nM, in the linear ranges from 0.01 to 7.0 μM and from 7.0 to 150 μM, using a sensor based on GCE modified by a core-shell nanocomposite and containing polypyrrole (PPy) as a core and ZIF-8 as a shell [176]. Moreover, quercetin determination in tablets and urine samples was achieved at a GCE modified by MoS_2_ nanoflowers and a 3D graphene aerogel (3D MoS_2_-GA) nanocomposite, which showed an LOD of 2.6 nM in the linear range from 0.01–5.0 μM [179]. Moreover, quercetin determination in adult human blood and urine, pregnant female blood, and human breast milk was determined using an SeO_2_/rGO/GCE sensor that showed an LOD of 1.6 nM in the linear range from 0.0 to 200 μM [159].

Rutin determination in human urine was achieved with: (i) a Gr/AuNPs/AN sensor with an LOD of 25 nM in the linear ranges from 0.08 to 10 μM and from 0.02 to 20 mM [187], and (ii) an MB@ZIF-8/rGO/GCE sensor with an LOD of 20 nM in the linear range from 0.1 to 100 μM [188]. The detection of rutin human serum was obtained using a N, S@C-dots-modified GCE, with an LOD of 0.8 nM in the linear range from 2 to 1300 nM, [186].

The flavonol galangin was electrochemically detected in human urine at a p-rGO/NAF/GCE sensor that showed an LOD of 1.11 nM in the linear range from 0.02 to 45 μM [109].

## 4. Conclusions

The review describes the fundamental principles underlying flavonoid redox behavior from the point of view of their antioxidant activity and discusses flavonoid oxidation pathways and the influence of the structure, substituents and experimental conditions. The latter sections focus on the electroanalysis of flavonoids in natural products and the challenges of identifying them in complex matrices such as fruits, vegetables, beverages, food supplements, nutraceuticals, and human body fluids.

The correct assessment of flavonoid redox properties is essential for promoting the applications of electrochemical methods in the routine quality control of food products, medicinal herbs and pharmaceutical drugs and supplements, where flavonoid content and antioxidant activity needs to be determined. Flavonoids present a common oxidation behavior that occurs at the hydroxyl groups linked to the aromatic rings, while the electroactive and non-electroactive substituents can also influence their voltammetric profile. Flavonoid antioxidant capacity is highly related to the number and conjugation of the hydroxyl groups present in their structure. Flavonoids with high antioxidant capacity present a high number of hydroxyl groups and low oxidation potentials, whereas those with high oxidation potentials can even act as pro-oxidants. A high oxidation peak current is normally related to a higher reaction rate and/or number of electrons transferred.

Over the past decade, electrochemical sensors have proved to be effective for the detection and quantification of flavonoids and other polyphenols in complex matrixes of biological samples. Due to their high sensitivity, specificity, low cost, easy miniaturization and use of small amounts of sample, they have become one of the first choices of many researchers and analysts.

In natural samples, flavonoids can occur in free forms, as glycosylated or acylated derivatives, or as oligomeric and polymerized structures, which affect their physicochemical behavior and complicate their electrochemical analysis. Prior to the phenolic compounds’ electrochemical identification and determination, natural samples generally require preliminary steps of separation and solvent extraction from the biological matrix. For this reason, it was not possible to develop a single standardized procedure for the determination of all flavonoid classes; different methodologies have been developed and optimized, depending on the nature of the sample and the target flavonoid analyte.

Another difficulty in flavonoid determination in natural matrixes consists of the superposition of the oxidation peaks of different phenolic compounds. Additional research is required to improve the simultaneous electroanalytical determination of various flavonoid classes in natural matrices, in order to prevent erroneous outcomes caused by their comparable structures and interfering substances.

Another key aspect to consider in the electrochemical determination of flavonoids is the choice of electrode material and sensor architecture. To enhance the electron transfer, to improve the sensor capture efficiency and to amplify the electrochemical response, various types of nanostructured materials have been used for the modification of the electrode surface of electrochemical sensors. Examples may include metal nanoparticles, nanorods, nanostructures, graphene, graphene oxide, reduced graphene oxide, quantum dots, carbon nanotubes, metal–organic frameworks, molecularly imprinted polymers and other polymers, aerogels, DNA, or dendrimers. Among them, the most promising results were obtained with electrochemical sensors based on nanocomposites and molecularly imprinted polymers. Nevertheless, further studies are needed to enhance flavonoid electroanalytical quantification, especially in biological fluids, where the flavonoid concentration is reduced, with most studies being based on flavonoid spike-and-recovery experiments.

## Figures and Tables

**Table 1:** Flavonoids classification and the major contributors to their dietary intake.

FLAVONOID SUBCLASS	FLAVONOIDS EXAMPLES	DIETARY SOURCE
FLAVONES 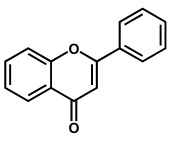	Apigenin, Baicalein, Chrysin, Diosmin, Diosmetin, Isorhoifolin, Linarin, Luteolin, Orientin, Tricin 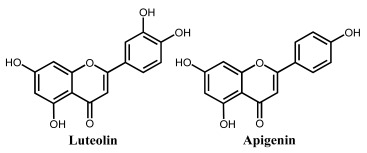	celery, olives, onion, lettuce, parsley, oregano, rosemary, thyme, green pepper, red wine, milk, chamomile tea, olive oil, peppermint oil, *Ginkgo biloba* etc.
FLAVANONES 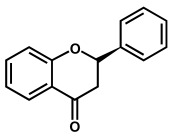	Eriodictyol, Hesperitin, Hesperidin, Naringenin, Naringin 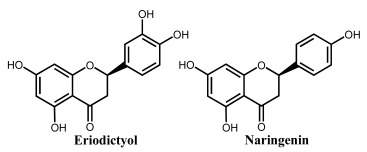	apples, lemon, lime orange, orange, grapefruit, tangerine, peppermint, etc.
FLAVONOLS 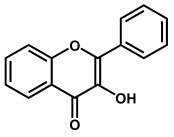	Fisetin, Galangin, Isorhamnetin, Kaempferol, Morin, Myricetin, Quercetin, Rhamnetin, Robinin, Rutin 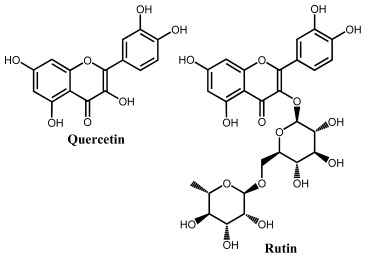	apples, apricots, grapes, plums, blackberries, blueberries, cranberries, currants, cherries, apple juice, *Ginkgo biloba*, onion, lettuce, capers, celery, dock leaves, fennel, hot peppers, cherry tomatoes, spinach, sweet potato leaves, turnip (green), endive, leek, lettuce, celery, broccoli, red wine, etc.
FLAVANONOLS 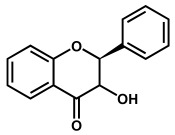	Dihydromyricetin, Taxifolin 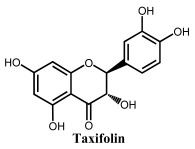	apples, citrus, etc.
FLAVANOLS 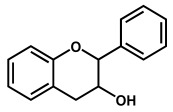	Catechin, Gallocatechin, Epicatechin, Epicatechin gallate, Epigallotechin gallate 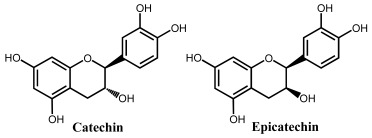	apples, apricots, grapes, peaches, nectarines, pears, plums, raisins, raspberries, cherries, blackberries, blueberries, and cranberries, red wine, tea leaves, coffee, cacao beans, etc.
ISOFLAVONOIDS 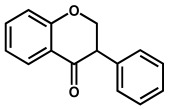	Biochanin, Daidzein, Equol, Genistein, Puerarin 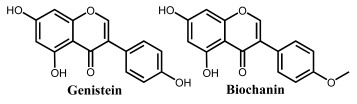	soy beans, fava beans, etc.
ANTHOCYANIDINS 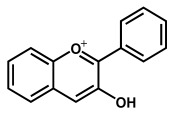	Cyanidin, Delphinidin, Malvidin, Peonidin 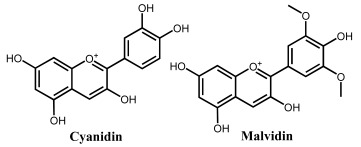	blackberries, blackcurrants, blueberries, black grape, elderberries, strawberries, cherries, plums, cranberry, pomegranate juice, and raspberry, red cabbage, red wine, etc.
ANTHOCYANINS 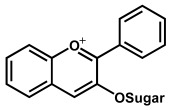	Cyanin, Kuromanin, Oenin, Malvin, Peonin 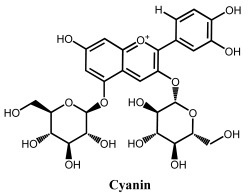	red fruits, grapes, red cabbage, purple sweet potato, *Tradescantia pallida* leaves, wine, etc.

**Table 2:** Flavonoids oxidation peak potentials, *E*_p_ vs. Ag/AgCl (3 M KCl), correlated with their substituent groups.

FLAVONOIDS		*E*_p_^1^/V	*E*_p_^2^/V	pH	SUBSTITUENTS							REF
					3^C^	5^A^	7^A^	2′	3′	4′	5′	
FLAVONES	Apigenin	0.68/0.82	0.99	4.3	H	OH	OH	H	H	OH	H	[24,25,26,27]
	Acacetin	–	0.98	4.3	H	OH	OH	H	H	OCH_3_	H	[25]
	Luteolin	0.40	1.05	4.0	H	OH	OH	H	OH	OH	H	[28,29,30,31]
	Orientin	0.25	0.83	7.0	H	OH	OH	O-sugar	OH	OH	O-sugar	[32]
	Diosmin	0.70	1.08	4.0	H	OH	O-sugar	H	OH	OCH_3_	H	[33,34]
	Diosmetin	0.68	1.06	4.0	H	OH	OH	H	OH	OCH_3_	H	[34]
	Isorhoifolin	0.65	1.10	5.0	H	OH	O-sugar	H	H	OH	H	[34]
	Linarin	–	1.10	5.0	H	OH	O-sugar	H	H	OCH_3_	H	[34]
	Chrysin	–	1.05	4.0	H	OH	OH	H	H	H	H	[24,35,36]
FLAVANONES	Eriodictyol	0.25	0.83	7.0	H	OH	OH	H	OH	OH	H	[32]
	Hesperitin	0.52	1.00	7.0	H	OH	OH	H	OH	OCH_3_	H	[37]
	Hesperidin	0.65	–	5.0	H	OH	O-sugar	H	OH	OCH_3_	H	[38]
	Naringenin	0.71	0.95	7.0	H	OH	OH	H	H	OH	H	[37]
	Naringin	0.39	–	5.0	H	OH	O-sugar	H	H	OH	H	[38,39]
FLAVONOLS	Myricetin	0.30	–	3.6	OH	OH	OH	H	OH	OH	OH	[38,40]
	Quercetin	0.20	0.4	7.0	OH	OH	OH	H	OH	OH	H	[37,38,41,42,43,44]
	Rutin	0.40	1.06	4.0	O-sugar	OH	OH	H	OH	OH	H	[24,38,45,46,47]
	Quercetrin	0.32	–	5.0	O-sugar	OH	OH	H	OH	OH	H	[38,48]
	Fisetin	0.35	–	5.0	OH	H	OH	H	OH	OH	H	[38,44,49,50]
	Rhamnetin	0.30	–	4.5	OH	OH	OCH_3_	H	OH	OH	H	[38]
	Isorhamnetin	0.36	1.15	4.0	OH	OH	OCH_3_	H	OH	OH	H	[44,51]
	Morin	0.44	0.98	4.0	OH	OH	OH	OH	H	OH	H	[24,44,52,53,54,55]
	Kaempferol	0.45	–	3.6	OH	OH	OH	H	H	OH	H	[40,44,56]
	Galangin	0.50	–	5.0	OH	OH	OH	H	H	H	H	[24,38]
	Robinin	0.75	0.95	7.0	O-sugar	OH	O-sugar	H	OH	H	H	[32]
FLAVANONOLS	Taxifolin	0.39	1.03	4.0	OH	OH	OH	H	OH	OH	H	[35]
FLAVANOLS	Catechin	0.28	0.61	7.0	OH	OH	OH	H	OH	OH	H	[37,57,58,59]
	Epicatechin	0.21	0.60	7.0	OH	OH	OH	H	OH	OH	H	[37]
	Epicatechin gallate	0.45	0.87	2.0	gall	OH	OH	H	OH	OH	H	[60]
	Epigallotechin gallate	0.38	0.89	2.0	gall	OH	OH	H	OH	OH	OH	[61]
ISOFLAVONOIDS	Genistein	0.48	0.67	7.0	H (2^C^)	OH	OH	H	H	OH	H	[25,26]
	Biochanin	–	0.69	7.0	H (2^C^)	OH	OH	H	H	OCH_3_	H	[26]
	Daidzein	0.50	0.72	7.0	H (2^C^)	H	OH	H	H	OH	H	[26]
ANTHOCYANIDINS	Cyanidin	0.40	0.82	4.5	OH	OH	OH	H	OH	OH	H	[62]
	Malvidin	0.49	0.85	4.5	OH	OH	OH	H	OCH_3_	OH	OCH_3_	[62]
	Peonidin	0.35	0.82	4.5	OH	OH	OH	H	OCH_3_	OH	H	[62]
	Petunidin	0.040	0.85	4.5	OH	OH	OH	H	OCH_3_	OH	OH	[62]

**Table 3:** Electroanalytical determination of selected flavonoids in complex matrixes. Flavonoids presented in alphabetical order (A-L entries).

FLAVONOIDS	SENSOR	METHOD	LINEAR RANGE	LOD	MATRIX	REF.
Apigenin	Ni NPs/SPE		0.9–200 μM	5 nM	Chinese medicine *Lobelia chinensis*	[27]
Baicalein	Bi_2_O_3_-CMWCNTs/GCE	LSV	0.01–15 μM	2.0 nM	Chinese medicine *Oroxylum indicum*; human urine	[85]
Baicalein	MoO_3_-PEDOT NWs	DPV	0.005–0.86 μM	1.5 nM	Chinese medicine *Scutellaria baicalensis Georgi*	[86]
Catechin	GCE	SWV	6.7–16.7 ppm	0.84 ppm	*C. mellei*; *C. quadrifidus*	[58]
Catechin	GPAC/GCE	CV; DPV	4–368 μM	0.67 μM	Green tea leaves	[87]
Catechin	SPE-CB/MoS_2_	DPV	0.12–25 μM	0.017 μM	Cocoa powder	[88]
Catechin	SPCE	A; BIA	1–150 μM	0.021 μM	Bioactive plant extracts	[89]
Catechin	Pt/AuNPs–PPy/Tyr	CV; A	1–10 nM	1.2 nM	Apple juice	[90]
Catechin	β-CD/CPE	SWV	––	1.35 mg/mL	Commercial tea	[57]
Catechin	pAF-GC	DPV	0.25 30 μM	72 nM.	Tea	[91]
Catechin	Asp/GC	DPV	0.25–30 μM	72 nM	Tea	[91]
Catechin	MWCNT/YHCF/GCE	CV	5–200	0.28 μM	Tea	[92]
Catechin	SWCNT-SubPc/GCE	DPV	0.1–1.5 μM	13 nM	Tea	[93]
Catechin	gallic acid/MWCNT/CPE	DPV-MCR-ALS	0.10–2.69 μM	0.017 μM	Green tea	[94]
Catechin	PDATT/Den(AuNPs)/laccase	CV; CA	0.1–10 μM	0.05 μM	Green tea	[95]
Catechin	Ni(II) complex–SAM-Au	SWV	3.31–25.3 μM	0.83 μM	Green tea	[96]
Catechin	TAT-PI-1/1	DPV	50–350 μM	15.2 μM	Green tea	[97]
Catechin	SWCNTs/ PEDOTM/GCE	CV; CC	0.039–40.84 μM	0.013 μM	Green tea	[98]
Catechin	CMC-CNT	CV	5–194 μM	0.06–0.12 μM	Green tea	[99]
Catechin	mMIPs/rGO-ZIF-8/GCE	DPV	0.01 nM–10 μM	0.003 nM	Green tea	[100]
Catechin	Pt/MnO_2_/f-MWCNT/GCE	CV; SWV	2–950 μM	0.02 μM	Green tea; black tea; red wine	[101]
Catechin	PMB/CPE	CV; DPV	0.1–1 μM; 1 μM–1.0 mM	4.9 nM	Green tea; fruit juice	[102]
Catechin	N-Gr/GCE	DPV	1.0–30 μM	0.088 μM	Chinese green tea	[103]
Catechin	I-GCE	Potentiometric titration	10–600 mM	0.6 mM	Wine	[104]
Catechin	laccase/CTS-g-N-CSIDZ-4- MBA@GNP/GD/GCE	CV; CA	0.8–16.6 μM	16 nM	Industrial sewage	[105]
Catechin	SWNTs-CTAB/GCE	CV; DPV	0.4–2.4 nM	0.1 nM	Standard	[106]
Daidzein	MWCNT/GCE	SWV	0.2–1.25 μM	80 nM	Standard	[70]
Dihydromyricetin	DNA-GCE	DPV,	0.04–2 μM	20 nM	Ampelopsis	[107]
Diosmin	GCE	AdS-DPV	0.05–9 μM	35 nM	Pharmaceutical	[33]
Epigallocatechin Gallate	GCE	SWV	0.1–1 μM	65.9 nM	Green tea	[61]
Fisetin	Au-BMI.PF_6_/Ni(II) complex/Si	SWV	0.28–1.39 μM; 2.77–19.50 μM	0.05 μM	Apple juice	[49]
Fisetin	CPT-BDD	SWV	1.7–6.9 μM	0.28 μM	Nutritional supplements	[108]
Galangin	p-rGO/NAF/GCE	DPV	0.02–45 μM	1.11 nM	Pharmaceutical; human urine	[109]
Genistein	HMD	SWV	0.1–1.1 μM	34 nM	Soy flour; soy supplement	[19]
Hesperidin	MIP/AuNPs/UAC@GCE	DPV	85 nM–30 μM	45 nM	Tangerine peel	[110]
Hesperidin	LIG chip	LSV	50 nM–0.1 mM	15 nM	Citrus fruit	[111]
Hesperidin	Polyaluminon/f-SWCNT/GCE	DPV	0.1–2.5 μM; 2.5–25 μM	20 nM	Orange, grapefruit juices	[112]
Hesperidin	MWCNT-BPPGE; MWCNT-SPE	CV; AdSV	up to 30 μM	0.61 μM; 7 nM	Orange juice; standard	[113]
Hesperidin	nGp-Bg/MCPE	DPV	0.1–7; 7–100 μM	50 nM	Fortified fruit juice	[114]
Hesperidin	BDD	AdSV	4.1 μM to 0.1 mM	1.2 μM	Dietary supplements	[64]
Hesperidin	SiO_2_/CPE	DPV	0.5–25 μM	0.25 μM	Traditional Chinese medicines	[115]
Hesperidin	Au NPs/rGO/GCE	A	0.005–8.0 μM	8.2 nM	Traditional Chinese medicines	[116]
Hesperidin	ePGE	DPV	0.5–10 μM	0.2 μM	Pharmaceutical	[117]
Kaempferol	MIL-100(Fe)-MWCNTs/PEDOT/GCE		50–1950 nM	13.2 nM	*XinDaKang* tablets	[118]
Luteolin	MOF-525/MPC/GCE	CV, DPV	5 nM–0.1 μM; 0.1–5 μM	0.35 nM	Human serum; urine; tomato, watermelon juices	[119]
Luteolin	NAF-AuNFsBPC/GCE	CV, DPV	0.15–1.8 μM; 1.8–10 μM	0.07 μM	Human urine	[120]
Luteolin	MOF-801/MC-3/GCE	DPV; CV	0.02–0.2 μM; 0.2–10 μM	2.90 nM	Urine; green tea	[121]
Luteolin	MWCNTs-BMIMPF_6_/GCE	DPV	5 nM–1 μM	0.5 nM	*Chrysanthemum*	[122]
Luteolin	PCV/MWCNTs/GCE	DPV	0.02–70 μM	5 nM	*Chrysanthemum*	[123]
Luteolin	Au/Pd/rGO/GCE	DPV	0.01─80.0 μM	0.98 nM	*Chrysanthemums*; peanut shells	[124]
Luteolin	MIP/MoS_2_/GNCNTs/GCE	LSV	0.04–2.0 μM	9.0 nM	Carrot; *Chrysanthemum* tea	[125]
Luteolin	MoO_3_-PPy NWs/ MWCNTs/GCE	DPV	0.1 nM–10 μM	0.03 nM	*Chrysanthemum* tea	[126]
Luteolin	MoO_3_-PEDOT/CD-MOF/GCE	DPV	0.0004–1.8 μM	0.1 nM	*Chrysanthemum* tea	[127]
Luteolin	MOF UiO-66/rGO/GCE	DPV	0.001–20 μM	0.75 nM	*Chrysanthemums*, hawthorn extracts	[128]
Luteolin	Co_3_O_4_@N-CNTs/NH_2_-GrQDs/GCE	DPV	0.5–1000 nM	0.1 nM	*Chrysanthemum* extracts	[129]
Luteolin	ZrO_2_/CS/rGOA-GCE	DPV; CV	5–1000 nM	1 nM	Red wine; peach juice	[130]
Luteolin	Ti_3_C_2_-MXene/ZIF-67/ CNTs/GCE	DPV	0.1–1000 nM	0.03 nM	Grape juice	[131]
Luteolin	BNNS-AuNPs/GCE	SWV	5–1200 pM; 0.02–10 μM	1.7 pM	Perilla; peanut hulls	[63]
Luteolin	Pt–BPC/CILE	CV, DPV	0.008–100 μM	2.6 nM	*Duyiwei* capsules	[132]
Luteolin	Fe_3_O_4_@MIP/rGO/GCE	CV, DPV	0.01–1 nM; 1 nM–50 μM	3 pM	*Duyiwei* capsules	[133]
Luteolin	MIP/ITO	DPV	0.05–30 μM	24 nM	Traditional medicine	[134]
Luteolin	HAP–CNT/GCE	DPV	40–12 μM	0.8 nM	Standard	[135]

Abbreviations provided at the end of Table 4.

**Table 4:** Electroanalytical determination of selected flavonoids in complex matrixes. Flavonoids presented in alphabetical order (M-Z entries).

FLAVONOIDS	SENSOR	METHOD	LINEAR RANGE	LOD	MATRIX	REF.
Morin	Gr/DMF/GCE	voltammetry	0.008–1	8.0 nM	Human serum	[136]
Morin	PEDOT–Au/rGO/GCE	SWV	1–150 mM	8.3 μM	Human serum	[137]
Morin	CPE-NiPc	DPV	10 μM–2.5 mM	2.0 nM	Human urine; *Psidium guajava*; red wine, tea, guava leaf capsules	[138]
Morin	V_2_O_5_NF/GCE	CV; DPV	0.05–10.93 μM	9 nM	Kiwi; Strawberry	[139]
Morin	rGO/[Co(NH_3_)_6_]^3+^/GCE	CV; DSP; EIS	0.008–72.35 μM	1.0 nM	Fruits	[140]
Morin	NH_2_-MWCNT/ZnO/SPCE	CV; DPV	0.2–803.4 μM	2 nM	Strawberry; avocado; mulberry leaves	[141]
Morin	CPB/SWNT-COOH/GCE	DPV	0.1–100; 100–750 μM	28.9 nM	Mulberry leaves	[142]
Morin	f-CNF/Tb_2_Se_2_/GCE	CV; DPV	2.5–158 μM	0.6 μM	Guava leaves	[143]
Morin	Ir–PEDOT/CFP	CV; DPV	0.12–2.80 nM	42.18 pM	Guava, mulberry leaves; grape wine	[144]
Morin	Pd-TCNSs(1000)/CFP	CV; DPV	37.5–130 pM	572 fM	Guava, mulberry leaves	[145]
Morin	β-CD-PANI/PGE	DPV	1.17–32 nM	0.38 nM	Mulberry leaves; almonds	[146]
Morin	NiTe_2_/CPE	CV; DPV	0.014–32 μM	13 nM	Red wines	[147]
Morin	MWCNTs/CCE	DPV; ASV	0.7–3 μM (DPV); 0.6–4 μM (ASV)	0.27 μM (DPV); 0.3 μM (ASV)	Tea	[148]
Morin	PGE	SWASV	0.1–1.33 μM	0.2 nM	Standard	[149]
Morin	PTFE-DNA/GCE	CV; LSV; CC	––	––	Standard	[150]
Naringin	LIG chip	LSV	50 nM–0.1 mM	11 nM	Citrus fruit	[111]
Naringin	Polyaluminon/f-SWCNT/GCE	DPV	0.1–2.5 μM; 2.5–25 μM	29 nM	Orange, grapefruit juices	[112]
Puerarin	CeO_2_N/CNT/GCE	A	0.04–6.0 μM	8 nM	Pharmaceutical	[151]
Quercetin	NPGF	CV	0.01–12; 12–100 μM	1.1 nM	Standard	[152]
Quercetin	DNA/CPE	DPV	0.1–3 μM	3.8 nM	Standard	[153]
Quercetin	CNTPE	CC; CV	2–100 nM; 0.1–20 μM	<2 nM	Rutin hydrolysate product	[154]
Quercetin	CTAB/CPT-BDD	SWV; AdSV	1.7 nM–0.33 μM	0.44 nM	Apple juice	[65]
Quercetin	Carbon-kaolin nanocomposite	CV, DPV	0.12–182.1 mM	0.057 mM	Apple juice	[155]
Quercetin	PB-rGO/TCD/AuNPs/GCE	DPV	0.005–0.4 μM	1.83 nM	Red wine; apple juice; honeysuckle	[156]
Quercetin	GCE	DPV	0.1–15 μM	3.1 nM	Apple, pear juices; red, green tea	[157]
Quercetin	Au/CeO_2_@FGCM–PE	CV, SWV	48 nM–1.09 μM	0.37 nM	Green tea; apple, grape juices; onion; honeysuckle	[158]
Quercetin	SeO_2_/rGO/GCE	A	0.0–200 μM	1.6 nM	Grape, apple, pear juices; honey; green, black tea; blood; breast milk; urine	[159]
Quercetin	g-C_3_N_4_/NiO/GCE	DPV	0.010–250 μM	2.0 nM	Green apple; green tea; honeysuckle	[160]
Quercetin	E-Gr/HP-β-CD/GCE	DPV	0.005–20 μM	1.0 nM	Tea; honeysuckle	[161]
Quercetin	NAF-CNT-GCE	O-SWV	0.02–2 μM	<0.2 μM	Yellow onion	[162]
Quercetin	AgNPs@gCN/GCE	DPV	0.1 nM–0.12 mM	6 nM	Green apple	[163]
Quercetin	ZnO/CNS/MCPE	DPV	0.17–3.63 μM	0.04 μM	Onion; honey buckwheat; standard	[164]
Quercetin	Au QDs/Au NP/GCE	DPV	0.01–6.0 μM	2.0 nM	Peanut hulls	[165]
Quercetin	3D SWCNTs-coumarin/GCE	DPSV	0.25–3 μM	20 nM	Tea	[166]
Quercetin	CPE/AM	DPV	0.025–1.5 μM	10 nM	Tea; honeysuckle	[167]
Quercetin	hp-Au/Au	CV; A	20 nM–100 μM	3.9 nM	Food; drinks; pharmaceutical	[168]
Quercetin	MrGO-MIP/SPE	DPV	20 nM–250 μM	13 nM	Pharmaceutical	[169]
Quercetin	HOPNC/GCE	DPV	0.1–20 μM; 20–120 μM	0.03 μM	*Ginkgo* tablet	[170]
Quercetin	Pt-Au-BPC/CILE	CV; DPV	0.15–6.0 μM; 10.0–25.0 μM	50.0 nM	*Ginkgo* tablet	[171]
Quercetin	GCE	CV	––	<100 nM	Onion bulbs	[172]
Quercetin	CNTPE	AdS-CV	0.1–1 μM	30 nM	Human serum; urine	[173]
Quercetin	CuWO4@PANI/GCE	DPV	0.001–0.500 μM	1.2 nM	Human blood; urine	[174]
Quercetin	WS_2_/GCE	DPV	10 nM–50 μM	2.4 nM	Human blood	[175]
Quercetin	PPy@ZIF-8/GCE	DPV	0.01–7.0 μM; 7.0–150 μM	7 nM	Human plasma	[176]
Quercetin	CoON-GCE	CC	0.50–330 μM	100 nM	Human urine; *Ginkgo* tablet	[177]
Quercetin	Au-b-CDs/NH2-GQDs/GCE	DPV	1–210 nM	285 pM	Human serum; honey; tea; honeysuckle	[178]
Quercetin	3D MoS_2_-GA/GCE	DPV	0.01–5.0 μM	2.6 nM	Tablets; urine	[179]
Quercetin; Rutin	CNTPE	DPV	0.05–5 μM; 0.10–10 μM	0.5 μM; 0.2 μM	Standard mixture	[180]
Rutin	IL-CPE	DPV	5–80 nM	<5 nM	Buckwheat seeds	[181]
Rutin	PSSA/MWCNTs/MBT/Au	DPV	0.01–0.8; 0.8–10 μM	1.8 nM	Red apple; red onion; oat; orange; strawberry; salvia	[182]
Rutin	CoFe_2_O_4_/GO/MBCPE	CV; DPV	0.001–0.1 μM; 1.0–100 nM	0.94 pM	Red apple, lime, lemon, orange juices	[183]
Rutin	ZnO-rGrO-PB/MCPE	CV; DPV	0.07–7.0 μM; 7.0–100 μM	20 nM	Fruit juice	[184]
Rutin	GCE/rGO/ZIF-8/MIP	DPV	0.0005–0.05 μM; 0.05–100 μM	0.1 nM	Orange juice; tablets	[185]
Rutin	N, S@C-dots/GCE	DPV	2–1300 nM	0.8 nM	Oranges; pharmaceutical tablets; human serum	[186]
Rutin	Gr/AuNPs/AN	DPV	0.08–10 μM; 0.02–20 mM	25 nM	Human urine; tablets	[187]
Rutin	MB@ZIF-8/rGO/GCE	CV; DPV	0.1–100 μM	20 nM	Human urine; tablets	[188]
Rutin	AgZA-CCE	CV; DPV; CA	0.005–0.21 μM	0.47 nM	*Capparis spinosa* extract; black tea	[189]
Rutin	CNTPE	DPV	0.2–10 μM	34 nM	Pharmaceutical	[190]
Rutin	Cu(II)-resin-carbon composite	CV	1–8 μM	26.5 nM	Pharmaceutical	[191]
Rutin	CNTPE	SWV	0.08–1.4 μM; 2.0–160.0 μM	50 nM	Pharmaceutical	[192]
Rutin	NiGO/GCE	CV; SWV	0.01–1 μM; 2.2–15 μM	3.2 nM	Pharmaceutical	[193]
Rutin	Gr-AuNPs/SPCE	SWV	0.1–15 μM	11 nM	Pharmaceutical	[194]
Rutin	Mg-Al-Si@PC/GCE	CV; DPV	1–10 mM	0.01 μM	Pharmaceutical	[195]
Rutin	LF-GCE	SWV	0.5–10 nM	0.25 nM	Tablets	[196]
Rutin	Au-AgNTs/NG	CV; DPV	0.1–420 μM	15 nM	Tablets	[197]
Rutin	ss-HGC	SWV	4 nM–1 μM	1 nM	Tablets	[198]
Rutin	BP–PEDOT: PSS/GCE	DPV	0.02–15 μM; 15.0–80 μM	7 nM	Tablets	[199]
Rutin	DNA-IL-CP	DPV	8 nM –10 μM	1.3 nM	Tablets	[200]
Rutin	CNTPE	DPV	48–960 μM	33.9 nM	Tablets	[201]
Rutin	PABSA-GCE	SWV	0.25–10 μM	100 nM	Tablets	[202]
Rutin	CB/WO_3_/SPCE	DPV	0.01–75.5 μM	2 nM	Tablets	[203]
Rutin	CoWO_4_/PC	DPV	5–5000 ng/mL	0.45 ng/mL	Tablets	[204]
Rutin	Al-MOF/MWCNT/GCE	DPV	1.0 nM–3.0 μM	0.67 nM	Tablets	[205]
Rutin	Co/ZIF-C/GCE	CV; DPV	0.1–30 μM	22 nM	Vitamin tablets	[206]
Rutin	meso-Co_3_O_4_/rGO/GCE	DPV	0.1–300 μM	0.03 μM	Standard	[207]
Taxifolin	SPE	SWV	0.05–1 μM	0.021 μM	Peanut oils	[208]
Taxifolin	E-rGO/GCE	AdSV	10 nM–1 μM	2 nM	*Polygoni Orientalis Fructus*	[209]
Taxifolin	PDDA-Gr-Pd/GCE	SWV	40 nM–1 μM	1 nM	*Polygoni Orientalis Fructus*	[210]
Taxifolin	rGO-Co_3_S_4_@MoS_2_/GCE	DPV	5 nM–1 μM	1.67 nM	*Polygoni Orientalis Fructus*	[211]
Taxifolin	MoS_2_/ANC/GCE	DPV	1 nM–1 μM	0.3 nM	*Polygoni Orientalis Fructus*	[212]
Taxifolin	Ni-MOF/CNTs	DPV	40 nM–10 μM	13 nM	*Polygoni Orientalis Fructus*	[208]
Taxifolin	MoS_2_/PPC/GCE	DPV	70 nM–10 μM	23 nM	Standard	[213]

Common abbreviations in Table 3 and Table 4: *γ*-cyclodextrin metal–organic framework(CD-MOF); 1-butyl-3-methylimidazolium hexafluorophosphate (BMIMPF6); 2-mercaptobenzothiazole (MBT); 2,4,6-triamino-1,3,5-triazine (TAT); 3′,4′- diamine-2,2′; 5′,2′′-terthiophene, (PDATT); acupuncture needle (AN); adsorption stripping voltammetry (AdSV); amperometry (A); alumina microfiber (AM); aspartic acid (Asp); Au-Ag nanothorns (Au-AgNTs/NG); Au NPs in an ionic liquid 1-butyl-3-methylimidazolium hexafluorophosphate (Au-BMI.PF6); batch injection analysis (BIA); beta-cyclodextrin (β-CD); biomass porous carbon (BPC); bismuth oxide-carboxylated MWCNs (Bi_2_O_3_-CMWCNTs); black phosphorene (BP); boron nitride nanosheets (BNNS); boron-doped diamond (BDD); carbon black (CB); carbon ceramic electrode (CCE); carbon fiber paper (CFP); carbon ionic liquid electrode (CILE); carbon nanotubes paste electrode (CNTPE); carbon paste electrode (CPE); carboxymethylcellulose (CMC); cationic cetylpyridium bromide (CPB); cathodically pre-treated BDD (CPT-BDD); cetylramethylammonium bromide (CTAB); dendrimer (Den); differential pulse stripping voltammetry (DPSV); electro-activated disposable pencil graphite electrode (ePGE); electro-deposited Gr (E-Gr); electrodeposited rGO (E-rGO); glassy carbon electrode (GCE); graphene (Gr); graphene oxide (GO); graphene sheet-like porous activated carbon (GPAC); graphitic carbon nitride (gCN); highly porous gold film modified gold electrode (hp-Au/Au); ionic liquid (IL); laser-induced graphene (LIG); lead film (LF); limit of detection (LOD); macroporous carbon (MPC); magnetic bar carbon paste electrode (MBCPE); magnetic molecularly imprinted polymers (mMIPs); magnetic rGO (MrGO); mercaptobenzoic acid (MBA); mesoporous carbon (MC); mesoporous Co_3_O_4_ nanospheres encapsulated with reduced graphene oxide (meso-Co_3_O_4_/rGO); metal–organic framework (MOF); Methylene Blue (MB); molecularly imprinted polymers (MIPs); MoS_2_ and N-doped active carbon composite (MoS_2_/ANC); MoS_2_-graphene aerogel (MoS_2_-GA); multiwalled carbon nanotubes (MWCNTs); N,Ndimethylformamide (DMF); N and S co-doped carbon dots (N, S@C-dots); nafion (NAF); nanoflakes (NFs); nano-graphene-platelet/Brilliant-green (nGp-Bg); nanoparticles (NPs); N-doped Gr (N-Gr); Ni-based MOF and CNTs composite (Ni-MOF/CNTs); nickel (II) phthalocyanine (NiPc); oxidized carbon nanofiber (f-CNF); pencil graphite electrode (PGE); poly (3,4–ethylenedioxythiophene) (PEDOT); poly(styrenesulfonate) (PSS); poly(crystal violet) (PCV); poly(diallyl dimethyl ammonium chloride) (PDDA); poly(hydroxymethylated-3,4-ethylenedioxythiophene) (PEDOTM); poly(p-aminobenzene sulfonic acid) (PABSA); poly(styrenesulfonate) (PSS); polyaniline (PANI); poly(crystal violet) (PCV); polyimide (PI); polymerized β-cyclodextrin (P-β-CD); polymerized MB (PMB); polypyrrole (PPy); poly(sulfosalicylic acid) (PSSA); porous carbon (PC); PC encapsulated Mg-Al-Si alloy (Mg-Al-Si@PC); porous N-doped graphene (N-Gr); pyrolytic graphite electrode (BPPGE); porous rGO (p-rGO); reduced graphene oxide (rGO); quantum dot (QD); screen printed carbon electrode (SPCE); screen-printed electrode (SPE); self-assembled monolayer (SAM); single walled carbon nanotubes (SWCNTs); single-sided heated graphite cylindrical (ss-HGC); thynyl bearing subphthalocyanine (SubPc); Touch me not (*Mimosa pudica*) carbon nano spheres (TCNSs); triaminotriazine (TAT); ultrafine electro-polymerized activated carbon (UAC); yttrium (III) hexacyanoferrate (YHCF); zeolite imidazolate frameworks (ZIF).
